# Consensus on items and quantities of clinical equipment required to deal with a mass casualties big bang incident: a national Delphi study

**DOI:** 10.1186/1471-227X-14-5

**Published:** 2014-02-22

**Authors:** Edward A S Duncan, Keith Colver, Nadine Dougall, Kevin Swingler, John Stephenson, Purva Abhyankar

**Affiliations:** 1 NMAHP Research Unit, Scion House, University of Stirling, Stirling, FK9 4LA, Scotland, UK; 2 Scottish Ambulance Service, Edinburgh, Scotland, UK; 3 School of Nursing Midwifery and Health, The University of Stirling, Stirling, Scotland, UK; 4 Department of Maths and Computing Science, University of Stirling, Stirling FK9 4LA, Scotland, UK; 5 National Ambulance Resilience Unit, Unit 9 Granada Trading Estate, Demuth Way, Oldbury B69 4LH, UK

**Keywords:** Mass casualties, Major incident, Ambulance, Delphi method, Internet, Paramedic, Big bang, Clinical equipment

## Abstract

**Background:**

Major short-notice or sudden impact incidents, which result in a large number of casualties, are rare events. However health services must be prepared to respond to such events appropriately. In the United Kingdom (UK), a mass casualties incident is when the normal response of several National Health Service organizations to a major incident, has to be supported with extraordinary measures. Having the right type and quantity of clinical equipment is essential, but planning for such emergencies is challenging. To date, the equipment stored for such events has been selected on the basis of local clinical judgment and has evolved without an explicit evidence-base. This has resulted in considerable variations in the types and quantities of clinical equipment being stored in different locations. This study aimed to develop an expert consensus opinion of the essential items and minimum quantities of clinical equipment that is required to treat 100 people at the scene of a big bang mass casualties event.

**Methods:**

A three round modified Delphi study was conducted with 32 experts using a specifically developed web-based platform. Individuals were invited to participate if they had personal clinical experience of providing a pre-hospital emergency medical response to a mass casualties incident, or had responsibility in health emergency planning for mass casualties incidents and were in a position of authority within the sphere of emergency health planning. Each item’s importance was measured on a 5-point Likert scale. The quantity of items required was measured numerically. Data were analyzed using nonparametric statistics.

**Results:**

Experts achieved consensus on a total of 134 items (54%) on completion of the study. Experts did not reach consensus on 114 (46%) items. Median quantities and interquartile ranges of the items, and their recommended quantities were identified and are presented.

**Conclusions:**

This study is the first to produce an expert consensus on the items and quantities of clinical equipment that are required to treat 100 people at the scene of a big bang mass casualties event. The findings can be used, both in the UK and internationally, to support decision makers in the planning of equipment for such incidents.

## Background

Major short-notice or sudden impact (known as big bang [1]) incidents which result in a large number of casualties are, fortunately, rare events. However they do occur and health services must be prepared to respond appropriately. In the United Kingdom (UK), as with most developed countries, normal response ambulances will not have the capacity to carry the extra equipment which is required to care for these patients while at the incident [2].

In order to deal with a big bang mass casualties incident, National Health Service (NHS) organizations, including ambulance services must be supported by extraordinary measures [1]. As part of their role UK NHS ambulance services maintain and deploy extra clinical equipment for big bang mass casualties emergencies [2]; and, on arrival at such an incident, establish and manage a casualty clearing station. Individuals are then triaged and receive emergency medical treatment as required before transportation to hospital. However, the London Assembly Report into the 2005 London Bombings highlights the challenges of achieving this in practice: The London Ambulance Service lacked essential supplies, such as fluids triage cards and tourniquets, at all sites [3].

Predicting the types and quantities of clinical equipment that will be required at a mass casualties big bang event is difficult. It is necessary to consider the wide range of incidents [1], both natural and man-made, that could cause such an event, and the resultant broad spectrum of potential clinical need:- e.g. haemorrhage, burns, respiratory disorders; fractures; effects of smoke inhalation etc. The response must also be tailored to the level of care that can be practically delivered in a pre-hospital environment. A recent systematic review highlighted the lack of evidence to inform policymakers and service providers about the types and quantities of clinical equipment required at a mass casualties big bang event [4].

Current UK ambulance service provision of clinical equipment at big bang mass casualties incidents has developed on the basis of local clinical judgment over many years, without any central co-ordination or clear evidence-base. This has resulted in variations in stock type and quantity throughout the UK. Agreeing the types and quantities of clinical equipment required at a big bang mass casualties emergency would be advantageous. At a national level it would provide policy and strategic decision-makers with knowledge to support them in planning future service provision. More locally it may enable services to make more efficient use of their resources.

This study, therefore, aims to address the current lack of knowledge about appropriate clinical equipment for dealing with a mass casualties big bang [1] event. Specific research questions are: what are a) the most important items of clinical equipment required to treat 100 people at the scene of a big bang mass casualties event?; and b) the minimum quantities required of each item?

## Methods

Participants were asked to consider what would be required to provide immediate patient care for 100 people in the pre-hospital phase of a big bang mass casualties incident. The study was based on current UK planning assumptions [1,5] for such events (Table 1). The figure of 100 people was chosen, firstly as it was a conceptually straightforward number of casualties to conceptualize, and secondly as it would allow easy calculations of quantities of items required at mass casualty incidents, as the results of the study could be simply multiplied as required.

**Table 1:** **Planning assumptions for the potential percentages of casualties in each category**[1,4]

**Category**	**Patient condition**	**% of total**
P1	Casualties needing immediate life-saving resuscitation and/or surgery	25% (25 casualties)
P2	Stabilized casualties needing early surgery but delay is acceptable	25% (25 casualties)
P3	Casualties requiring treatment but a longer delay is acceptable	50% (50 casualties)

A modified Delphi study method was used. Originally developed by the RAND Corporation in the 1950’s [6], the Delphi method has since been used extensively in healthcare research [7-11], including emergency care research [12-17], amongst other fields. Since its inception, many Delphi studies have varied slightly from the original RAND Corporation method, and it is therefore common to find studies described as ‘modified Delphi studies’ , or using a Delphi approach [7]. Delphi studies use a form of consensus methodology to develop a reliable consensus of a group of experts on a specific topic. The Delphi method involves a series of questionnaires, or ‘rounds’ (typically 3), on a specific topic being completed by subject experts. These rounds are interspersed by controlled feedback which includes the participant’s own judgment and the overall group judgment for comparison. Participants are then given the opportunity to revise their judgment in the following round if they so desire. Participants’ individual responses are unknown to the group [18].

Given the variability of study methods that have been used and described as ‘Delphi’, it is important to outline the features that ensure the credibility of findings for this approach. These are: a clear description of why a Delphi method has been used; the choice of participants that form the expert panel; transparency of data collection procedures used; the choice of consensus level; and the means of dissemination [19]. A study reference group comprised of a small number of key leaders in the field was formed to support the study. Key tasks for the group were to: agree the study protocol; identify potential participants; provide expert comment on the study findings.

An opinion on the status of this study was sought from the NHS Lothian Ethics Committee who advised that for the purposes of ethical approval, the study was classifiable as a service evaluation [20]. The Scottish Ambulance Service Research Governance Committee, as the NHS Scotland Special Health Board for pre-hospital emergency care, granted Research and Development study approval. All data and participant information was stored securely in line with good research practice guidelines.

### Sample

Participants were purposively selected according to the following criteria, which defined our ‘expert participant’:-

1. Individual clinical (paramedical or medical) experience of providing a professional pre-hospital emergency medical response to a mass casualties incident; or

2. Responsibility in health emergency planning for mass casualties’ incidents and be in a position of authority and influence within the sphere of health emergency planning and response.

Potential participants were identified through the study reference group and researchers’ knowledge base. The researchers used a snowballing method of recruitment to increase the potential participant base by asking the initial group to identify other potential participants who met the inclusion criteria. Letters of invitation to participate in the study were sent to 141 individuals. The majority of people invited to participate were located in the UK, but a few (n = 7) were based in other countries with similar emergency response strategies.

People interested in participating in the study were asked to email the study research paramedic (KC) to note their interest. They were then provided with a unique password, log-in, and link to the study website. The password and log-in linked the individual in each round of data collection, and enabled them to exit and re-enter the study website in order to complete each round as their time allowed.

### Data collection

Data was collected using a purposively designed study website. This enabled the study to be carried out on-line via a web browser instead of relying on paper-based questionnaires. Although the website was developed specifically for this study, it was designed in a manner that would allow its use in further Delphi studies with minimal adaptation. Individuals could not register and take part in the study from the site alone – they needed the password and unique identifier that was sent to them by the research team. Inter-round data analysis was completed automatically and significantly reduced the administration that is normally required to be undertaken between rounds of a Delphi study. The web site included the usual features you would expect from such a service. Having logged in, the user was presented with the Delphi questionnaire and, if they had already started it, the values they had entered. Participants were prompted to save their responses as they progressed through the study and whenever they logged out of the website. This allowed the participant to return to the site and complete the questionnaire in more than one sitting. Electronic reminders were sent automatically two weeks after the commencement of each round, and also in the final stages to those individuals who had not yet completed the round. These reminders stated the final date by which the current round must be completed. An a priori decision was made to limit the study to three rounds of data collection to minimize participant fatigue [21]. The website was piloted for acceptability and usability by Scottish Ambulance Service Special Operations Response Team ambulance clinicians and emergency planning officers. Feedback from the pilot stage was positive, although individuals noted that the task was substantial due to the number of items included.

#### Round one

Items for round one (n = 232) were collated from the researchers’ existing knowledge of current stock for mass casualties incidents in the UK. The list of items to rate was long, so they were split into subsets according to their purpose (i.e. Items relating to Airway; Breathing; Circulation; Examination Medicines; Splintage; Comfort; Control of Infection; Transport; Other) each with a separate tab on the web-page. This made the questionnaire look less daunting and helped users find the item they had reached if they had saved their partial progress, and returned later.

Participants were asked to carry out two tasks for each listed item. Firstly, they were asked to rate the importance they would give to each item along a scale of 1 to 5 (Very unimportant – 1; Quite unimportant – 2; Neither – 3; Quite important – 4; Very important - 5); and secondly, they were asked to state how much of each item they believed would be required to treat 100 patients at the scene of a big bang mass casualties incident. Participants were offered the chance to click a button to declare that they had no opinion or knowledge for any given item. This also allowed an automatic check via the web site that no items had been accidently missed. The web site displayed a bar to inform the user of their progress and offered a facility to help them find any items they had missed. Participants who had completed less than 100% of the questionnaire were automatically emailed a reminder before the end of each round. Participants were also able to add any clinical items (for inclusion in round two) which they felt were important but missing from the round one list.

#### Round two

Participants were asked to review the aggregated findings for the previous round together with their previous individual ratings, as well as 16 unique items of clinical equipment added in round one. Participants were invited to reconsider their rating of importance and quantification for each item. As in the previous round, electronic reminders were sent out to all non-completing participants after two weeks.

#### Round three

Participants were again asked to review the aggregated findings for the previous round together with their previous individual ratings, and were again invited to reconsider their rating and quantification for each item of equipment in respect of the results of round two. Electronic reminders were sent out after two weeks.

### Data handling

**Figure 1:**
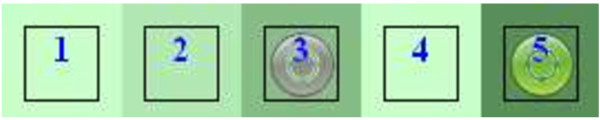
An example from the website of a color histogram of previous responses.

Delphi studies vary considerably in how they handle and analyze their data [18]. The computerization of the study allowed the data to be presented to participants in a novel and more meaningful way. Data from rounds two and three were presented to participants as a color histogram (or heat map) where the depth of color indicated the frequency with which respondents in the previous round had chosen each rating. Figure 1 shows the frequency with which each of the five responses had been chosen in the previous round (dark being many, light being few). The grey circle shows the choice that the current participant made on the previous round and the green circle shows the choice that they have made on the current round, (in round one each box was white as no previous selection had been made). In this way, participants could easily see how their responses compared to the consensus in the previous round and either confirm or update their response accordingly.

The second question required a numeric answer. As the user sample size in each round exceeded 30 (and therefore the number of independent responses was sufficient to assume that the central limit theorem held with responses tending towards being normally distributed), we proceeded to adopt a parametric approach in the iterative feedback to users between rounds. Feedback to the user was given as a color again, but in this case, the depth of color indicated the number of standard deviations between the user’s response and the mean response (in other words, the z-score). As the scale for each answer was different, the normalized z-score provided a consistent measure of agreement for each question. Z-scores were calculated as,

$$z=\frac{x-\mu }{\sigma }$$

**Figure 2:**
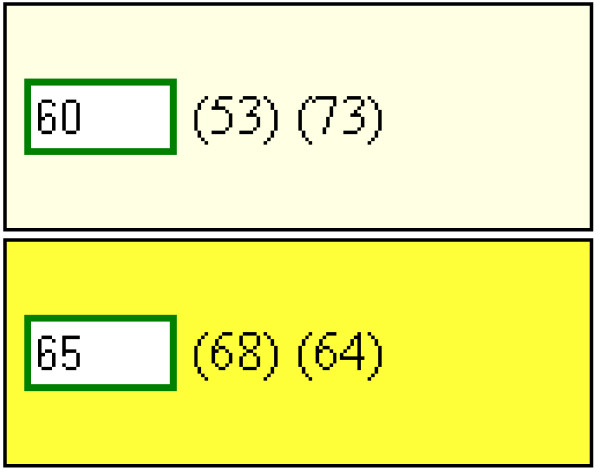
The quantity input box for two items as presented on the website.

Where *x* was the value for which the z-score is to be calculated, μ was the mean of the values of the previous round and σ was the standard deviation of the values from the previous round. The z-score was translated into a color depth and shown around the input box for each item in the questionnaire. The mean value from the previous round, along with the participant’s own response from the previous round were also displayed on the questionnaire. An example of the quantity input box is given in Figure 2; the top box shows that the previous average quantity for this item was 73 and that this participant had said 53. The light color indicates the difference. The bottom box shows where the participant was in closer agreement in the last round. The numbers in the boxes show the participant’s updated response for this round.

### Consensus

As the focus of this study was to understand which items should be included in a mass casualties response, it was important to understand which items gained consensus as being ‘important’ or ‘very important’, and conversely, which items were viewed as being ‘unimportant’, or ‘very unimportant’. An item was deemed to be ‘important’ or ‘very important’ if it had been rated as either four or five by at least 80% of respondents. Similarly, an item was deemed to be ‘unimportant’ or ‘very unimportant’ if it had been rated as either two or one by at least 80% of respondents.

### Analysis plan

Frequently in Delphi studies the mean value and standard deviation of ratings are presented. However, these are likely to be sub-optimal measures as it is more likely that the responses will form a skewed distribution. For example, if half the respondents in our study chose a score of 1, and half chose 5, then reporting a mean of 3 would fail to illustrate that the data had a bi-modal distribution. Therefore we proposed use of non-parametric approaches in the data analyses.

Research Question 1: A descriptive analysis of the total number of items that reached consensus of being important (agreement by at least 80% of participants) would be summarized. A statistical test of significant difference in consensus of item importance between rounds would be tested by Wilcoxon Rank Sum Test for independent events - if the 20 additional items added to the list between rounds one and two achieved consensus; otherwise the Wilcoxon Signed Rank Test for matched pairs was proposed.

Research Question 2: A descriptive analysis of the recommended median quantities of items that reached consensus (agreement by at least 80% of participants) would be summarized. A statistical test of significant difference in consensus of median items required between rounds will be tested by a Wilcoxon Signed Ranks Test for independent events.

## Results

Sixty individuals responded to the letter of invitation stating that they wished to participate. Forty-five participants (75%) completed round one of the study. Thirty-five participants (58%) completed round two, and 32 participants (53%) completed round three; 16 were ambulance clinicians, and 16 were medical personnel.

### Item consensus

A total of 16 new items were added following round one. Raters gained consensus on one hundred and thirty four items (54%) by round three. This figure increased to 164 items (66%) if the items which the raters neared consensus on (those > = 70%) are considered. Almost all the items which reached consensus were viewed as ‘important’ or ‘very important’ by participants; only four items on which raters reached consensus on were viewed as ‘unimportant’ or ‘very unimportant’ (i.e. rectal thermometer; Clopidogrel (300 mg); Clindamycin; Saline ampule (5 mls)); a further two items ‘nearing’ consensus, that is reaching 70-79% agreement, were also rated as ‘unimportant’ or ‘very unimportant’ (i.e. OPA (Size 000); ET Tube size 10).

There was considerable variation in the percentage of items that gained consensus within the subsets that had been split according to each item’s purpose. Subsets with the highest percentage of items reaching consensus were ‘Control of Infection’ (100%; n = 10); ‘Splintage’ (73%; n = 8); and ‘Circulation’ (73%; n = 35). Subsets that had the fewest items that reached consensus were ‘Medicine2’ (31%; n = 5); and ‘Airways2’ (35%; n = 11). Consensus for each of these subsets increased when items ‘nearing’ consensus (i.e. >70%) were also considered, (see Table 2). Items that gained consensus amongst raters as being important or very important are listed in Table 3. A full list of items and levels of consensus reached is also provided, (see Additional file 1).

**Table 2:** Consensus levels of items’ importance

**Group**	**Number of items in group**	**Agreement of number of items at end of round 3 (%)**		
		**≥ 80%**	**≥ 70%**	**≤ 69%**
Airway 1	17	10 (59)	12 (71)	5 (29)
Airway 2	31	11 (35)	15 (48)	16 (52)
Breathing	16	10 (62)	10 (62)	6 (38)
Circulation	48	35 (73)	44 (92)	4 (8)
Examination	18	9 (50)	9 (50)	9 (50)
Medicine 1	50	21 (42)	31 (62)	19 (38)
Medicine 2	16	5 (31)	7 (44)	9 (56)
Splintage	11	8 (73)	10 (91)	1 (9)
Comfort	6	3 (50)	3 (50)	3 (50)
COI	10	10 (100)	10 (100)	0 (0)
Other	14	6 (43)	6 (43)	8 (57)
Transport	11	6 (43)	7 (64)	4 (36)
**Totals**	**248**	**134 (54)**	**164 (66)**	**84 (34)**

**Table 3:** Items that gained consensus as ‘Important’ and ‘Very Important’ and their recommended quantities

**Subset & item (shaded items neared consensus)**	**Quantity**
	**Median (IQR)**
**Airway 1**	
OPA size 3	25 (20–25)
OPA size 4	25 (20–25)
Suction catheter - hard	30 (25–35)
Nasopharyngeal airway 7	22 (2–25)
Suction - handheld manual	23 (20–25)
Non-inflatable SG Airway device	21 (20–25)
Nasopharyngeal airway 6	22 (20–25)
OPA size 2	15 (10–15)
Particulate respirators [dust mask]	75 (50–100)
Suction - battery powered	10 (8–10)
**Airway2**	
ET Securing device	25 (20–25)
Catheter mount	25 (25–25)
Laryngeal mask size 2	10 (5–10)
Laryngeal mask size 4	16 (15–20)
Laryngoscope & Blade(s) - adult	20 (15–20)
Magill forceps - adult	20 (15–20)
ET Tube size 8	15 (10–15)
Surgical airway set	8 (5–10)
ET Tube size 7	15 (10–15)
Laryngeal mask size 5	15 (10–15)
Laryngoscope & Blade(s) - child	10 (6–10)
**Breathing**	
Bag Valve mask - adult	25 (20–25)
Bag Valve mask - child	10 (10–13)
Chest seal	25 (25–30)
Reservoir mask and tubing - adult	50 (40–50)
Reservoir mask and tubing - child	25 (25–30)
Nebuliser mask and tubing - adult	25 (24–30)
Nebuliser mask and tubing - child	20 (15–20)
Portable ventilator	10 (10–15)
Chest/thoracic drainage kits and sets	15 (10–15)
Bag valve mask - infant	10 (5–10)
**Circulation**	
3 way tap & extension tube (IO/IV)	35 (30–40)
Blast bandage	80 (75–100)
Disposable latex free tourniquet IV access	50 (50–50)
EZ IO	10 (10–10)
Haemostatic dressings	80 (70–100)
Administration set blood and blood derivatives	55 (50–60)
Combat application tourniquet	60 (50–70)
Syringe 2 ml	50 (46–50)
Transpore/micropore tape	55 (50–60)
Cannula 16 g	50 (50–50)
Cannula 18 g	50 (50–51)
IO Catheter	30 (25–36)
IO needle blue	15 (14–20)
IO needle red	15 (10–20)
IO needle yellow	15 (12–20)
Cannula 20 g	50 (40–50)
Dressing - extra large	90 (75–100)
Dressing - large	100 (100–100)
Dressing - medium	100 (100–110)
Dressing wound with conforming stretch bandage	85 (80–100)
IV Dressing - Transparent	100 (100–125)
Oales modular bandage	60 (50–95)
Swabs packet	95 (76–100)
Cling film - one roll	29 (20–30)
Hypodermic needles 19 g	50 (50–50)
Syringe 5 ml	50 (50–60)
Dressing burn gel-soaked sterile	62.5 (50–75)
AED	6 (5–10)
Safety/Drawing up needle	100 (93–100)
Cannula 22 g	30 (25–40)
Hypodermic needles 25 g	50 (50–50)
Syringe 1 ml	20 (15–25)
Syringe caps male/female red	100 (80–100)
AED Pads - adult	12 (10–15)
AED Pads - child	7 (5–10)
**Examination**	
Stethoscope - adult	20 (15–20)
Monitor SPO2	25 (22–30)
Glucose test strips (for use with glucose meter)	60 (50–75)
Triage tape - child	10 (10–14)
Glucose meter	10 (10–10)
Rectal thermometer	5 (0–5)
Monitor CO2	15 (10–20)
Torch - examination pen disposable	25 (25–25)
Manual sphygmomanometer	15 (10–15)
**Medicines1**	
Morphine sulphate	100 (84–100)
Oxygen mass delivery [1 unit]	3 (2–4)
Saline .9% 500 mls bag	90 (75–100)
Naloxone hydrochloride (Min-I-jet, 2 mg/5 mls)	35 (25–50)
Salbutamol - Nebuliser liquid 2 mg/ml 2.5 ml UDV	45 (40–50)
Clopidogrel 75 mg	2.5 (0–5)
Oxygen D size	28 (25–30)
Entonox [with mouthpiece]	25 (20–25)
Adrenaline (1 mg/1 ml, 1:1000)	20 (20–25)
Adrenaline (Min-I-Jet, 1 mg in 10 ml, 1:10,000)	40 (30–50)
Lidocaine -100 mg/10 ml (1%) solution for injection pfs	20 (15–25)
Saline ampoule 10 mls	100 (82–100)
Clopidogrel 300 mg	5 (0–5)
Ipratopium bromide (6 × 250 mcg/1 ml)	20 (15–20)
Clindamycin	5 (0–5)
Atropine sulphate (Min-I-Jet, 3 mg in 10 ml)	28 (25–31)
Diazepam emulsion 10 mg in 2 mls	22(20–25)
Eyewash 500 mls	30 (25–40)
Saline 10 mls pre-filled syringe	50 (50–52)
Saline ampule 5 mls	15 (0–20)
Oxygen F size	10 (10–12)
**Medicines2**	
Tranexamic acid	50 (40–50)
Ketamine	47.5 (40–50)
Midazolam	45 (35–50)
Suxamethonium chloride -Injection PFS 100 MG/2 ML	21 (20–25)
Ondansetron 4 mg	20 (19–25)
**Splintage**	
Pelvic sling	25 (25–30)
Cervical collars (set)	25 (20–30)
Head hugger with straps	25 (20–25)
Frac straps/packs	20 (16–25)
Traction splint	20 (16–20)
Triangular bandage	50 (50–50)
Box splint	25 (20–25)
Tape 100% cotton for medical or general use	25 (20–30)
**Comfort**	
Emergency blanket	120 (100–127)
Re-robe/modesty suits	65 (50–75)
De-robe suits	65 (50–75)
**Control of infection**	
Clinical waste bag	100 (100–120)
Latex free gloves extra large	102 (100–147)
Latex free gloves large	150 (107–195)
Latex free gloves medium	150 (139–195)
Latex free gloves small	115 (100–145)
Alcohol hand gel	50 (40–60)
Sharps box .2 lt	20 (14–20)
Sharps box 10 lt	10 (5–12)
Skin wipes (Tub)	30 (16–34)
Pre injection swabs	150 (112–200)
**Other**	
Entonox mouthpiece	50 (40–50)
Tuffcut scissors	30 (25–30)
Self help packs	75 (64–94)
Medication/drug syringe stickers	120 (100–150)
Shears - hardened stainless steel, circa 25 cm	20 (20–25)
Scalpel	25 (20–25)
**Transport**	
Carry sheet	30 (25–39)
Rescue board and straps	15 (10–20)
Stretcher - drag	15 (10–15)
Stretcher - orthopaedic	11 (10–15)
Stretcher - large wheeled	10 (4–10)
Stretcher - basket	10 (5–10)

Round one contained 232 items and rounds two and three contained an additional 16 items, bringing the total number of items to 248 for the last two rounds. The median Likert scores observed for 232 items in each of rounds one, two and three were 4, 5 and 5, respectively, (all with IQR 3 to 5). The non-parametric test for independent events failed to compute, most of the responses were tied in pairs, therefore the Wilcoxon Signed Ranks Test was used to assess whether there was a significant increase in consensus between rounds. A significant difference was found between median Likert scores for the 232 matched pairs of items between round one and round three (Z = -5.26; 151 ties; p < 0.001); but not between round two and round three (Z = -1.79; 215 ties; p = 0.074).

### Quantity of items required

The median quantities, and Inter Quartile Ranges (IQR), of items that gained consensus by raters are listed in Table 3. Whether the recommended quantities of items between rounds was a statistically significant improvement in participants’ consensus was tested using a Wilcoxon Signed Ranks Test. A significant difference was found between the median number of items for the 232 matched pairs of items between round one and round three (Z = -9.83; ties = 80; p < 0.001); and also between round two and round three (Z = -2.39; ties = 160; p = 0.017). Whilst participants suggested similar quantities for many items by round three, other items still had considerably wider recommended quantities. This is clearly evident in the persistently large IQR of some items (e.g. Large Latex Free gloves had a median recommend quantity of 150, but an interquartile range of 107–195).

## Discussion

Providing appropriate quantities of the right clinical equipment to the scene of a mass casualties big bang event is vital. But planning for such emergencies is challenging. This study has, for the first time, produced an expert consensus on the items and quantities of clinical equipment that are required to treat 100 people at the scene of a big bang mass casualties event. The results of this study clearly identify the equipment that is deemed of greatest importance. Items of clinical equipment that are highly likely to be required in the immediate care and treatment of trauma patients (e.g. morphine, oxygen, stretchers and carry sheets) were rated of greater importance than other items which may not be as essential (e.g. Hydrocortisone, Aspirin, Maternity pack, or Pillows).

The study used a specifically developed website for data collection. This enabled data to be presented to participants visually, overcoming some of the known limitations of using measures of central tendency [7,18,21,22] when feeding back results to participants between rounds. The web-based platform also reduced the length of time required to conduct the study: analysis of each round’s findings occurred automatically at a time appointed by the researchers and participants were immediately able to commence a further round of data collection. As data was stored electronically, the likelihood of human inputting error was also low and preliminary data analysis occurred automatically. However use of a web-based platform did not increase rates of study participation. Given its advantages, the researchers are now exploring the potential use of the web-based platform in future Delphi studies.

Consensus is reached in a Delphi study when a pre-agreed percentage of participants have rated items similarly. Ironically, there is little agreement within the Delphi study literature as to what constitutes a ‘correct’ percentage level of consensus. Previous Delphi studies show marked variation (between 50% - 100%) in consensus levels [18,23]. A consensus level of 80% was selected in this study as it marked a clear majority opinion and has been used in previous emergency medicine research using the Delphi method [14]. However, in recognition that the 80% cut-off selected was a strict and somewhat arbitrary definition of consensus, the items that gained at least 70% were highlighted in the study findings as items that were nearing the pre-set cut off. Ultimately the decision as to what is a sufficient degree of consensus is taken by those who use the study findings within their specific context, and not by the research team themselves.

In mass casualty big bang scenarios emergency equipment is transported to the scene as quickly as possible. The logistics of achieving this are considerable and space and resources are limited. This study provides policy makers and planners with information to support them in making an informed decision about the type and quantity of equipment that is immediately required at the scene to supplement the equipment, consumables and drugs that will already be on the ambulance vehicles attending the incident. The study results will help to minimize wastage from unnecessary equipment ordering, and to increase efficiency in routine equipment audits. The study findings were presented, at a UK wide implementation meeting, to a stakeholder group that comprised senior ambulance service clincians and managers, consultant emergency physicians, representatives from NHS England, NHS Wales and NHS Scotland, as well as English and Scottish Government policymakers and advisers. Structured discussions took place regarding the study findings. Services within NHS Scotland have since agreed to reconfigure their service and adopt the items that reached consensus, and their quantities, as the basis for stockpiling clinical equipment for mass casualties big bang incidents. In England and Wales, the output from the study and the implementation meeting have been presented to the NHS England Clinical Reference Group for Emergency Preparedness and are being used to redesign the contents of the English mass casualty vehicles.

### Limitations

Delphi studies are onerous tasks, both in terms of activity required and the duration of involvement. Consequently participant attrition is a known limitation, but often hard to accurately calculate as the numbers of people invited to participate are rarely reported. In this study only 32 (23%) of people invited to participate agreed and completed data collection over the three rounds. The study required participants to complete two tasks, a rating of an item’s importance, and a quantification of how many of each item was required, instead of one. The former task is one for which Delphi method is well suited and traditionally employed; the latter arguably less so. This dual task and the sheer number of items to be rated (n = 248) may have contributed to participant attrition during the study. Whilst this is disappointing, the actual numbers of participants who completed all three rounds (n = 32) is similar to other Delphi pre-hospital emergency care research [14,16]. Researchers undertaking future Delphi studies in pre-hospital emergency care should aim to keep the number of items as manageable as possible, and estimate that they will need to invite approximately five times more individuals than the number they wish to participate. Attaching two rating tasks per item, whilst feasible, is undesirable due to the potential negative effect this may have on participant retention.

Only 54% of items reached consensus by the end of the study. This figure may have improved had further rounds of data collection occurred, but as the consensus between rounds two and three was not statistically significantly different, it is not very likely. In any case, the potential benefits of further data collection had to be balanced against the potential of participant fatigue and the potential of decreasing response rates [21]. Delphi studies accept participant responses at face value. As elsewhere [17] this study would benefit from further qualitative investigation to understand participants’ responses in greater depth.

Whilst the median quantities of items are a useful starting point for services planning their resources, these need to be considered together with the local context that the service is working in: urban settings may have a higher frequency of standard emergency ambulances with a base loading of equipment, whereas more rural environments will have different considerations and require to factor in the longer distances that patients will be travelling to hospital following treatment on scene. Variation in the number of items required may have resulted from some participants misunderstanding the instructions, and consequently listing how many of an item they would wish to bring to an incident, rather than the number of items that they felt would actually be required on-scene.

## Conclusion

An expert panel of individuals with either clinical experience of providing a professional pre-hospital emergency medical response to a mass casualties incident or responsibility and authority in health emergency planning for mass casualties incidents reached a consensus that 134 items of emergency clinical equipment were either important or very important when responding to a big bang mass casualties event. A further 30 items neared the agreed 80% consensus level. Indicative quantities for each item were provided. The study findings provide an important resource for the UK, and other countries with similar response mechanisms and planning assumptions, to inform the development of evidence-based policies and the planning of future emergency responses to big bang mass casualties events.

## Competing interests

The authors declare that they do not have any financial or non-financial competing interests in relation to this paper.

## Authors’ contributions

EASD led the study design, managed the overall study, chaired the study advisory committee, and drafted and revised the manuscript; KC was the study research fellow, he conceived the study idea, undertook day-to-day study management, participated in the study advisory committee and contributed to revisions of the final paper; ND provided statistical advice and expertise, and commented on revisions to the final paper; KS designed and managed the study website and background database, and commented on revisions to the final paper provided read and approved of the final manuscript; JS provided expert policy awareness, convened the study advisory committee, and commented on revisions to the final paper; PA advised on study design and statistical analysis, participated in the study advisory committee and contributed to revisions of the final paper. All authors read and approved the final manuscript.

## Pre-publication history

The pre-publication history for this paper can be accessed here:

http://www.biomedcentral.com/1471-227X/14/5/prepub

## Supplementary Material

Additional file 1 Levels of consensus on all items and recommended quantities. Click here for file
