# Supplementary material for: Consensus on items and quantities of clinical equipment required to deal with a mass casualties big bang incident: a national Delphi study
Source: BMC Emerg Med. 2014 Feb 22;14:5. doi: 10.1186/1471-227X-14-5 (PMC3936839; doi:10.1186/1471-227X-14-5)
Supplement: Additional file 1 — Levels of consensus on all items and recommended quantities. [file 1471-227X-14-5-S1.docx]

**Additional File 1 – Levels of consensus on all items and recommended quantities.**

| **Subset & Item** | **CONSENSUS** | | **QUANTITY** | |
| --- | --- | --- | --- | --- |
|  | **Importance Rating**  **(Highest pairs)** | **% Agree**  **Pair** | **Median (IQR)** | |
| **AIRWAY 1** | | | | |
| OPA size 3 | 4 or 5 | 100 | 25 (20-25) | |
| OPA size 4 | 4 or 5 | 100 | 25 (20-25) | |
| Suction catheter - hard | 4 or 5 | 100 | 30 (25-35) | |
| Nasopharyngeal airway 7 | 4 or 5 | 93.55 | 22 (2-25) | |
| Suction - handheld manual | 4 or 5 | 93.55 | 23 (20-25) | |
| Non-inflatable SG Airway device | 4 or 5 | 91.67 | 21 (20-25) | |
| Nasopharyngeal airway 6 | 4 or 5 | 90.32 | 22 (20-25) | |
| OPA size 2 | 4 or 5 | 87.10 | 15 (10-15) | |
| Particulate respirators [dust mask] | 4 or 5 | 87.10 | 75 (50-100) | |
| Suction - battery powered | 4 or 5 | 87.10 | 10 (8-10) | |
| OPA size 1 | 4 or 5 | 74.19 | 10 (10-14) | |
| OPA size 000 | 1 or 2 | 70.97 | 5 (3-6) | |
| Nasopharyngeal airway 8 | 4 or 5 | 64.52 | 15 (10-20) | |
| Suction catheter - soft | 4 or 5 | 58.06 | 50 (25-50) | |
| Nasopharyngeal airway 9 | 1 or 2 | 48.39 | 10 (1-10) | |
| OPA size 00 | 1 or 2 | 41.94 | 5 (5-10) | |
| OPA size 0 | 4 or 5 | 19.35 | 8 (5-10) | |
| **AIRWAY 2** | | | | |
| ET Securing device | 4 or 5 | 93.55 | | 25(20-25) |
| Catheter Mount | 4 or 5 | 90.32 | | 25 (25-25) |
| Laryngeal Mask size 2 | 4 or 5 | 90.32 | | 10 (5-10) |
| Laryngeal Mask size 4 | 4 or 5 | 90.32 | | 16 (15-20) |
| Laryngoscope & Blade(s) - Adult | 4 or 5 | 90.32 | | 20 (15-20) |
| Magill Forceps - adult | 4 or 5 | 90.32 | | 20 (15-20) |
| ET Tube size 8 | 4 or 5 | 87.10 | | 15 (10-15) |
| Surgical airway set | 4 or 5 | 80.77 | | 8 (5-10) |
| ET Tube size 7 | 4 or 5 | 80.65 | | 15 (10-15) |
| Laryngeal Mask size 5 | 4 or 5 | 80.65 | | 15 (10-15) |
| Laryngoscope & Blade(s) - Child | 4 or 5 | 80.65 | | 10 (6-10) |
| Introducer | 4 or 5 | 77.42 | | 15 (11-17) |
| ET Tube size 10 | 1 or 2 | 74.19 | | 3 (0-5) |
| Magill Forceps - child | 4 or 5 | 74.19 | | 10 (5) |
| Gum elastic bougie | 4 or 5 | 73.08 | | 15 (5-10) |
| ET Tube size 6 | 4 or 5 | 67.74 | | 10 (10-18) |
| ET Tube size 9 | 4 or 5 | 64.52 | | 10 (5-10) |
| Stylet - adult | 4 or 5 | 56.67 | | 10 (5-15) |
| ET Tube size 5.5 | 1 or 2 | 54.84 | | 5 (2-8) |
| ET Tube size 8.5 | 1 or 2 | 51.61 | | 10 (0-10) |
| Laryngeal Mask size 3 | 4 or 5 | 51.61 | | 15 (10-15) |
| Magill Forceps - infant | 1 or 2 | 51.61 | | 5 (5-10) |
| Heat moisture exchanger without sampling port | 1 or 2 | 50.00 | | 10 (5-15) |
| ET Tube size 3 | 1 or 2 | 48.39 | | 5 (5-10) |
| ET Tube size 4.5 | 1 or 2 | 48.39 | | 5 (5-8) |
| Tube tracheotomy blue line | 4 or 5 | 46.43 | | 10 (5-12) |
| Stylet - child | 4 or 5 | 43.33 | | 9 (5-10) |
| ET Tube size 4 | 1 or 2 | 41.94 | | 5 (5-8) |
| ET Tube size 5 | 1 or 2 | 29.03 | | 5 (5-10) |
| Tube ryle nasogastric | 1 or 2 | 27.59 | | 10 (10-14) |
| Laryngeal Mask size 1 | 4 or 5 | 22.58 | | 9 (5-10) |
| **BREATHING** | | | | |
| Bag Valve Mask - adult | 4 or 5 | 100 | | 25 (20-25) |
| Bag Valve Mask - child | 4 or 5 | 100 | | 10 (10-13) |
| Chest seal | 4 or 5 | 100 | | 25 (25-30) |
| Reservoir Mask and tubing - adult | 4 or 5 | 100 | | 50 (40-50) |
| Reservoir Mask and tubing - child | 4 or 5 | 100 | | 25 (25-30) |
| Nebuliser mask and tubing - adult | 4 or 5 | 93.55 | | 25 (24-30) |
| Nebuliser Mask and tubing - child | 4 or 5 | 93.55 | | 20 (15-20) |
| Portable ventilator | 4 or 5 | 86.67 | | 10 (10-15) |
| Chest / thoracic drainage kits and sets | 4 or 5 | 86.21 | | 15 (10-15) |
| Bag Valve Mask - infant | 4 or 5 | 80.65 | | 10 (5-10) |
| Nasal cannula | 1 or 2 | 64.52 | | 10 (7-13) |
| Venturi | 1 or 2 | 54.84 | | 10 (5-11) |
| Simple facemask and tubing (oxygen) - adult | 4 or 5 | 38.71 | | 25 (20-25) |
| Simple facemask and tubing (oxygen) - child | 4 or 5 | 38.71 | | 15 (11-20) |
| CBRN filters for Bag Valve Mask | 4 or 5 | 35.48 | | 20 (11-25) |
| Inhaler spacer - child | 1 or 2 | 23.33 | | 5 (5-10) |
| **CIRCULATION** | | | | |
| 3 way tap & extension tube (IO/IV) | 4 or 5 | 100 | | 35 (30-40) |
| Blast bandage | 4 or 5 | 100 | | 80 (75-100) |
| Disposable Latex Free Tourniquet IV Access | 4 or 5 | 100 | | 50 (50-50) |
| EZ IO | 4 or 5 | 100 | | 10 (10-10) |
| Haemostatic dressings | 4 or 5 | 100 | | 80 (70-100) |
| Administration set blood and blood derivatives | 4 or 5 | 96.77 | | 55 (50-60) |
| Combat application tourniquet | 4 or 5 | 96.77 | | 60 (50-70) |
| Syringe 2 ml | 4 or 5 | 96.77 | | 50 (46-50) |
| Transpore/micropore tape | 4 or 5 | 96.77 | | 55 (50-60) |
| Cannula 16g | 4 or 5 | 96.67 | | 50 (50-50) |
| Cannula 18g | 4 or 5 | 96.67 | | 50 (50-51) |
| IO Catheter | 4 or 5 | 96.67 | | 30 (25-36) |
| IO needle blue | 4 or 5 | 96.67 | | 15 (14-20) |
| IO needle red | 4 or 5 | 96.67 | | 15 (10-20) |
| IO needle yellow | 4 or 5 | 96.67 | | 15 (12-20) |
| Cannula 20g | 4 or 5 | 93.55 | | 50 (40-50) |
| Dressing - extra large | 4 or 5 | 93.55 | | 90 (75-100) |
| Dressing - large | 4 or 5 | 93.55 | | 100 (100-100) |
| Dressing - medium | 4 or 5 | 93.55 | | 100 (100-110) |
| Dressing wound with conforming stretch bandage | 4 or 5 | 93.55 | | 85 (80-100) |
| IV Dressing -Transparent | 4 or 5 | 93.55 | | 100 (100-125) |
| Oales Modular Bandage | 4 or 5 | 93.55 | | 60 (50-95) |
| Swabs packet | 4 or 5 | 93.55 | | 95 (76-100) |
| Cling film - one roll | 4 or 5 | 90.32 | | 29 (20-30) |
| Hypodermic needles 19g | 4 or 5 | 90.32 | | 50 (50-50) |
| Syringe 5 ml | 4 or 5 | 90.32 | | 50 (50-60) |
| Dressing burn gel-soaked sterile | 4 or 5 | 90.00 | | 62.5 (50-75) |
| AED | 4 or 5 | 87.10 | | 6 (5-10) |
| Safety/Drawing up needle | 4 or 5 | 87.10 | | 100 (93-100) |
| Cannula 22g | 4 or 5 | 83.87 | | 30 (25-40) |
| Hypodermic needles 25g | 4 or 5 | 83.87 | | 50 (50-50) |
| Syringe 1 ml | 4 or 5 | 83.87 | | 20 (15-25) |
| Syringe caps male/female red | 4 or 5 | 83.33 | | 100 (80-100) |
| AED Pads - adult | 4 or 5 | 80.65 | | 12 (10-15) |
| AED Pads - child | 4 or 5 | 80.65 | | 7 (5-10) |
| Cannula 14g | 4 or 5 | 79.31 | | 50 (40-50) |
| Dressing absorbent cellulose with fluid repellent backing sterile | 4 or 5 | 79.31 | | 70 (50-77) |
| 2.5cm bandage | 4 or 5 | 78.57 | | 50 (25-50) |
| Pack sterile eye care | 4 or 5 | 77.42 | | 25 (20-30) |
| Syringe 20ml | 4 or 5 | 77.42 | | 50 (50-59) |
| Pad eye self adhesive | 4 or 5 | 76.67 | | 30 (25-36) |
| Steristrips (packet) | 4 or 5 | 74.19 | | 25 (20-25) |
| Hub cap for Syringe Luer Tip Rubber strip of 10 | 4 or 5 | 71.43 | | 30 (20-30) |
| Hypodermic needles 30g | 4 or 5 | 70.97 | | 45 (26-50) |
| Syringe 10ml | 1 or 2 | 64.52 | | 100 (100-100) |
| Syringe 50 ml | 4 or 5 | 61.29 | | 25 (20-30) |
| Nasal tampons | 4 or 5 | 43.33 | | 18 (10-20) |
| Plasters assorted (box of100) | 1 or 2 | 35.48 | | 10 (8-15) |
| **EXAMINATION** | | | | |
| Stethoscope - adult | 4 or 5 | 100 | | 20 (15-20) |
| Monitor SPO2 | 4 or 5 | 93.55 | | 25 (22-30) |
| Glucose test strips (for use with glucose meter) | 4 or 5 | 93.33 | | 60 (50-75) |
| Triage tape - child | 4 or 5 | 90.32 | | 10 (10-14) |
| Glucose meter | 4 or 5 | 90.00 | | 10 (10-10) |
| Rectal thermometer | 1 or 2 | 88.00 | | 5 (0-5) |
| Monitor CO2 | 4 or 5 | 83.87 | | 15 (10-20) |
| Torch - examination pen disposable | 4 or 5 | 83.87 | | 25 (25-25) |
| Manual Sphygmomanometer | 4 or 5 | 80.65 | | 15 (10-15) |
| Ear pieces for tympanic thermometer | 4 or 5 | 64.52 | | 75 (50-100) |
| Tympanic Thermometer | 4 or 5 | 64.52 | | 10 (5-10) |
| Auriscope | 1 or 2 | 50.00 | | 5 (5-5) |
| Auroscope ear pieces | 4 or 5 | 50.00 | | 30 (20-47) |
| Stethoscope - child | 1 or 2 | 22.58 | | 5 (5-10) |
| PEFR measure - adult | 1 or 2 | 20.00 | | 5 (5-6) |
| PEFR measure - child | 1 or 2 | 20.00 | | 5 (5-6) |
| Tongue depressor | 4 or 5 | 19.35 | | 35 (25-50) |
| PEFR tubes disposible | 1 or 2 | 16.00 | | 27 (20-30) |
| **MEDICINES 1** | | | | |
| Morphine Sulphate | 4 or 5 | 100 | | 100 (84-100) |
| Oxygen Mass delivery [1 unit] | 4 or 5 | 100 | | 3 (2-4) |
| Saline .9% 500mls bag | 4 or 5 | 100 | | 90 (75-100) |
| Naloxone Hydrochloride (Min-I-jet,2mg/5mls) | 4 or 5 | 96.55 | | 35 (25-50) |
| Salbutamol - Nebuliser liquid 2mg/ml 2.5ml UDV | 4 or 5 | 96.55 | | 45 (40-50) |
| Clopidogrel 75mg | 1 or 2 | 96.43 | | 2.5 (0-5) |
| Oxygen D size | 4 or 5 | 93.55 | | 28 (25-30) |
| Entonox [with mouthpiece] | 4 or 5 | 93.33 | | 25 (20-25) |
| Adrenaline (1mg/1ml, 1:1000) | 4 or 5 | 93.10 | | 20 (20-25) |
| Adrenaline (Min-I-Jet, 1mg in 10ml, 1:10,000) | 4 or 5 | 93.10 | | 40 (30-50) |
| Lidocaine -100mg/10ml (1%) solution for injection pfs | 4 or 5 | 92.59 | | 20 (15-25) |
| Saline ampoule 10 mls | 4 or 5 | 89.66 | | 100 (82-100) |
| Clopidogrel 300mg | 1 or 2 | 89.29 | | 5 (0-5) |
| Ipratopium Bromide (6 x 250mcg/1ml) | 4 or 5 | 89.29 | | 20 (15-20) |
| Clindamycin | 1 or 2 | 88.89 | | 5 (0-5) |
| Atropine Sulphate (Min-I-Jet, 3mg in 10ml) | 4 or 5 | 86.21 | | 28 (25-31) |
| Diazepam Emulsion 10mg in 2 mls | 4 or 5 | 86.21 | | 22(20-25) |
| Eyewash 500mls | 4 or 5 | 83.87 | | 30 (25-40) |
| saline 10mls pre-filled syringe | 4 or 5 | 82.76 | | 50 (50-52) |
| Saline ampule5 mls | 1 or 2 | 82.76 | | 15 (0-20) |
| Oxygen F size | 4 or 5 | 80.65 | | 10 (10-12) |
| Lubricating Jelly | 4 or 5 | 79.31 | | 25 (20-25) |
| Paracetamol -Tablets 500mg, 16 per pack | 4 or 5 | 79.31 | | 20 (20-25) |
| Water for injections (10ml) Ampoules | 4 or 5 | 79.31 | | 60 (50-75) |
| Paracetamol 1g IV | 4 or 5 | 76.00 | | 30 (25-30) |
| Cefotaxime (glass vial 500mg) | 4 or 5 | 74.07 | | 20 (19-23) |
| Dextrose 10% 500mls bag | 4 or 5 | 72.41 | | 15 (10-16) |
| Paracetamol -120mg/5ml oral suspension 5ml sachets sugar free | 4 or 5 | 72.41 | | 20 (19-25) |
| Water satches | 4 or 5 | 72.41 | | 50 (50-75) |
| Salbutamol-100mcg/dose MDI | 4 or 5 | 71.43 | | 14.5 (10-15) |
| Eyewash 20mls | 4 or 5 | 70.97 | | 32 (20-50) |
| Amiodarone Hydrochloride (300mg in 10ml) | 4 or 5 | 65.52 | | 15 (10-20) |
| Atropine sulphate 1mg in 10ml | 4 or 5 | 62.07 | | 20 (20-25) |
| Paracetamol fast melt tablets (250mg) | 4 or 5 | 62.07 | | 25 (20-30) |
| Saline 50ml bag | 1 or 2 | 62.07 | | 10 (7-15) |
| Calcium chloride | 4 or 5 | 51.85 | | 12 (10-15) |
| EMLA (tubes) | 1 or 2 | 42.86 | | 10 (5-10) |
| Benzylpenicillin Sodium (600mg) Ampoules | 4 or 5 | 39.29 | | 20 (6-23) |
| Oral Glucose Gel (40%) dextrose | 1 or 2 | 39.29 | | 10 (5-11) |
| Diazepam Rectal Solution, foil wrap (10 mg) | 4 or 5 | 34.48 | | 10 (10-10) |
| Diazepam Rectal Solution, foil wrap (5 mg) | 4 or 5 | 31.03 | | 10 (10-10) |
| Glucose - Oral Gel tube 23g, 3 tubes per pack | 4 or 5 | 31.03 | | 12 (10-15) |
| Glucose 10% 500mls | 4 or 5 | 31.03 | | 15 (10-15) |
| Suscard Buccal/Glycerol Trinitrate (2mg) | 4 or 5 | 31.03 | | 16 (10-20) |
| Chloramphenicol - Eye ointment 1% 4g | 4 or 5 | 25.93 | | 20 (19-25) |
| Glucagon (1mg/1ml) | 4 or 5 | 23.33 | | 10 (10-10) |
| Chlorpherinamine (10mg in 1ml) | 4 or 5 | 22.22 | | 10 (10-15) |
| Hydrocortisone (100mg in 1ml) Ampoules | 4 or 5 | 21.43 | | 15 (10-20) |
| Aspirin (300mg) | 4 or 5 | 17.24 | | 28 (25-30) |
| Metoclopramide HCL (10mg in 2 ml) ampoules | 4 or 5 | 14.29 | | 25 (20-25) |
|  | | | | |
| **MEDICINES 2** | | | | |
| Tranexamic Acid | 4 or 5 | 100 | | 50 (40-50) |
| Ketamine | 4 or 5 | 96 | | 47.5 (40-50) |
| Midazolam | 4 or 5 | 91 | | 45 (35-50) |
| Suxamethonium chloride -Injection PFS 100MG/2ML | 4 or 5 | 81 | | 21 (20-25) |
| Ondansetron 4mg | 4 or 5 | 80 | | 20 (19-25) |
| Rocuronium - 50m/5ml ampoule | 4 or 5 | 76 | | 30 (20-45) |
| Dicobalt Edetate | 4 or 5 | 70 | | 20 (10-25) |
| Co-Amoxiclav | 1 or 2 | 62 | | 12 (0-15) |
| Pancuronium 4mg | 1 or 2 | 56 | | 20 (5-22) |
| Beclomethasone | 1 or 2 | 48 | | 5 (0-5) |
| Fentanyl | 4 or 5 | 33 | | 20.5 (14-25) |
| Propafol | 4 or 5 | 33 | | 15 (10-15) |
| Cyclizine | 4 or 5 | 18 | | 20 (19-25) |
| Flumazenil | 4 or 5 | 18 | | 10 (10-10) |
| Proxymetacaine -0.5% eye drops 0.5ml unit dose preservative free | 4 or 5 | 18 | | 25 (20-30) |
| Sodium Bicarbonate | 4 or 5 | 17 | | 10 (10-10) |
| Splintage |  |  | |  |
| Pelvic sling | 4 or 5 | 96.77 | | 25 (25-30) |
| Cervical Collars (set) | 4 or 5 | 93.55 | | 25 (20-30) |
| Head Hugger with straps | 4 or 5 | 93.55 | | 25 (20-25) |
| Frac straps/packs | 4 or 5 | 90.32 | | 20 (16-25) |
| Traction splint | 4 or 5 | 90.32 | | 20 (16-20) |
| Triangular Bandage | 4 or 5 | 90.32 | | 50 (50-50) |
| Box splint | 4 or 5 | 87.10 | | 25 (20-25) |
| Tape 100% cotton for medical or general use | 4 or 5 | 80.65 | | 25 (20-30) |
| Extrication Device | 4 or 5 | 77.42 | | 10 (6-11) |
| Vacuum splint sets | 4 or 5 | 74.07 | | 15 (10-15) |
| Moldable foam splint | 4 or 5 | 69.23 | | 20 (15-20) |
| **COMFORT** | | | | |
| Emergency blanket | 4 or 5 | 96.77 | | 120 (100-127) |
| Re-robe/modesty suits | 4 or 5 | 96.77 | | 65 (50-75) |
| De-robe suits | 4 or 5 | 90.32 | | 65 (50-75) |
| Pillows | 1 or 2 | 54.84 | | 16 (10-20) |
| Earplugs | 1 or 2 | 38.71 | | 50 (25-72) |
| Survival bag | 4 or 5 | 35.48 | | 20 (11-25) |
| **CONTROL OF INFECTION** | | | | |
| Clinical waste bag | 4 or 5 | 100 | | 100 (100-120) |
| Latex free gloves extra large | 4 or 5 | 100 | | 102 (100-147) |
| Latex free gloves large | 4 or 5 | 100 | | 150 (107-195) |
| Latex free gloves medium | 4 or 5 | 100 | | 150 (139-195) |
| Latex free gloves small | 4 or 5 | 100 | | 115 (100-145) |
| Alcohol hand gel | 4 or 5 | 96.77 | | 50 (40-60) |
| Sharps Box .2 lt | 4 or 5 | 96.77 | | 20 (14-20) |
| Sharps Box 10 lt | 4 or 5 | 93.55 | | 10 (5-12) |
| Skin Wipes (Tub) | 4 or 5 | 93.55 | | 30 (16-34) |
| Pre injection swabs | 4 or 5 | 90.32 | | 150 (112-200) |
| **OTHER** | | | | |
| Entonox mouthpiece | 4 or 5 | 100 | | 50 (40-50) |
| Tuffcut scissors | 4 or 5 | 96.77 | | 30 (25-30) |
| Self help packs | 4 or 5 | 93.55 | | 75 (64-94) |
| Medication/drug syringe stickers | 4 or 5 | 93.33 | | 120 (100-150) |
| Shears - hardened stainless steel, circa 25cm | 4 or 5 | 90.00 | | 20 (20-25) |
| Scalpel | 4 or 5 | 89.66 | | 25 (20-25) |
| Incontinence Pads | 4 or 5 | 48.39 | | 50 (33-57) |
| Scalpel , blade size 15 | 1 or 2 | 44.83 | | 18 (11-20) |
| Large scalpel | 1 or 2 | 44.00 | | 10 (10-19) |
| Forceps | 4 or 5 | 34.48 | | 20 (15-20) |
| Maternity pack | 1 or 2 | 32.26 | | 4 (2-5) |
| Sanitary Towels | 1 or 2 | 16.67 | | 20 (10-23) |
| Ribbon gauze | 4 or 5 | 16.13 | | 20 (15-23) |
| Razor | 4 or 5 | 6.45 | | 20 (20-24) |
| **TRANSPORT** | | | | |
| Carry sheet | 4 or 5 | 100 | | 30 (25-39) |
| Rescue board and straps | 4 or 5 | 96.77 | | 15 (10-20) |
| Stretcher - drag | 4 or 5 | 90.00 | | 15 (10-15) |
| Stretcher - orthopaedic | 4 or 5 | 87.10 | | 11 (10-15) |
| Stretcher - large wheeled | 4 or 5 | 83.33 | | 10 (4-10) |
| Stretcher - basket | 4 or 5 | 80.65 | | 10 (5-10) |
| Stretcher - NATO | 4 or 5 | 77.42 | | 20 (16-25) |
| Basket stretcher cover | 4 or 5 | 64.52 | | 10 (5-10) |
| Rescue board - floating | 1 or 2 | 64.52 | | 5 (1-5) |
| Vacuum mattress | 4 or 5 | 61.29 | | 10 (5-10) |
| Glide sheet | 1 or 2 | 41.94 | | 10 (6-10) |
